# Combination of CEUS and MRI for the diagnosis of periampullary space-occupying lesions: a retrospective analysis

**DOI:** 10.1186/s12880-019-0376-7

**Published:** 2019-09-02

**Authors:** Xin-Pei Chen, Jiang Liu, Jing Zhou, Peng-Cheng Zhou, Jian Shu, Lu-Lu Xu, Bo Li, Song Su

**Affiliations:** 1 Department of Hepatobiliary Surgery, The Affiliated Hospital of Southwest Medical University, No. 25 Taiping Street, Jiangyang District, Luzhou, 646000 Sichuan Province China; 2 Department of Nephrology, The Affiliated Hospital of Southwest Medical University, Luzhou, 646000 Sichuan Province China; 3 Department of Ultrasonics, The Affiliated Hospital of Southwest Medical University, Luzhou, 646000 Sichuan Province China; 4 Department of Radiology, The Affiliated Hospital of Southwest Medical University, Luzhou, 646000 Sichuan Province China

**Keywords:** Magnetic resonance imaging (MRI), Contrast-enhanced ultrasound (CEUS), CCMW, Periampullary space-occupying lesions, Periampullary cancer

## Abstract

**Background:**

The value of magnetic resonance imaging (MRI), contrast-enhanced ultrasound (CEUS), and the combination of CEUS and MRI (CCWM) for the diagnosis of periampullary space-occupying lesions (PSOL) was investigated.

**Methods:**

A total of 102 patients diagnosed with PSOLs by surgery or biopsy were recruited retrospectively. The sensitivity, specificity, positive predictive value (PPV), negative predictive value (NPV), and accuracy of MRI, CEUS, and CCWM were analyzed.

**Results:**

MRI, CEUS, and CCWM allowed for the accurate detection of 91.17, 92.15, and 99.01% of PSOLs, respectively. The specificity, PPV, and accuracy of CCWM were significantly different from MRI and CEUS (*p* < 0.05). However, there the sensitivity and NPV were not significantly different among the three diagnostic technologies. In addition, the specificity, PPV, and accuracy were not significantly different between MRI and CEUS (all *p* > 0.05).

**Conclusions:**

CCWM is valuable for differentiating benign and malignant PSOL, which provides important guiding significances for the clinic.

## Background

A periampullary space-occupying lesion (PSOL) appears near the entrance of the common bile and pancreatic ducts, commonly within 2 cm of the ampullary of Vater. There are several kinds of PSOLs, including cholesterol stones, malignant and benign tumors, and inflammatory stenosis [1]. Periampullary cancer (PAC) arises within 2 cm of the major papilla in the duodenum and includes ampullary carcinomas, distal bile duct cancers, pancreatic head and uncinate process carcinomas, and duodenal carcinomas. PAC accounts for 0.5–2.0% of gastrointestinal malignancies diagnosed each year [2]. PAC is the most malignant type of cancer and surgical resection is only curative treatment, yet most patients are not surgical candidates [3]. After surgical resection, the average 5-year survival rates for patients with pancreatic adenocarcinomas and ampullary carcinomas are 17–25% and 21–38%, respectively [4]. If the PSOL is suspected to be malignant, an early expanded resection of the lesion is recommended by most physicians. In contrast, conservative treatment or endoscopic sphincterotomy should be considered for benign lesions, such as those caused by papillitis and papillary stenosis [5]. Therefore, the clinical detection and differentiation of benign and malignant PSOLs are essential for selecting the optimal treatment plan.

Currently, there are no specific symptoms or laboratory markers to quickly and accurately diagnose patients with benign or malignant PSOLs. Diagnostic imaging modalities, including magnetic resonance imaging (MRI) and contrast-enhanced ultrasound (CEUS), have been successfully used for the detection and diagnosis of PSOLs [6]. MRI is the preferred method for evaluating PSOLs due to its high soft-tissue resolution and lack of ionizing irradiation. Additionally, advanced MRI techniques, including magnetic resonance cholangiopancreatography (MRCP), dynamic contrast-enhanced MRI (DCE-MRI), and diffusion-weighted imaging (DWI), have been shown to enhance the detection sensitivity of PSOLs in the clinic [7–9]. However, there are some disadvantages of MRI that limit its clinical utility. For example, MRI is limited by its long acquisition times due to the high number of sequences required for image clarity [10]. In addition, MRI requires a magnetic field that is contradicted for patients with cardiac pacemakers, metal clips, or metal stents [8]. Previously, the specificity and accuracy of MRI for PSOLs were found to be 78.3 and 91.5%, respectively [6, 11].

Recently, real-time CEUS was used for the detection and diagnosis of PSOLs with an injection of SonoVue [12–14]. CEUS functions by assessing microbubble-enhanced blood flow and distribution. The primary advantages of CEUS include its high temporal resolution, lack of ionizing radiation, and real-time imaging capabilities [15]. However, CEUS is not preferred for imaging of PAC as its specificity and accuracy are inferior to MRI [6, 15]. Moreover, CEUS has some limitations, including its dependence on the operator experience and inability to visualize tissues at depths greater than 3 cm [16].

In this study, we explore the combination of MRI and CEUS, known as CCMW, for the detection of PSOLs. We aimed to demonstrate the usefulness of multimodality imaging in comparison to single-modality imaging in patients with PSOLs.

## Methods

### Study population

This was a retrospective study approved by the Institutional Review Board and Ethics Committee of the Affiliated Hospital of Southwest Medical College and written informed consent was obtained from all patients. A total of 102 patients who underwent CEUS and MRI examinations in the Department of Hepatobiliary and Pancreatic Surgery of the Affiliated Hospital of Southwest Medical University were enrolled from June 1, 2015 to May 1, 2018. All patients were diagnosed with PSOLs by pathology. The diagnostic results of the CEUS, MRI, and CCWM were compared with pathology to calculate the diagnostic accuracy of each method. For CCWM, if the malignant lesions were detected by CEUS or MRI correctly pre-surgery, the diagnosis of CCWM was defined as correct. For the benign lesions, preoperative diagnosis based on both CEUS and MRI had to be benign for the diagnosis to be recognized as accurate.

Inclusion criteria were as follows: (a) patients with pathologically-confirmed PAC; (b) patients with periampullary inflammatory lesions, including chronic mass-forming type pancreatitis, common bile duct (CBD) inflammatory stenosis, and duodenum papilla inflammation, all diagnosed by biopsy with follow-up more than 6 months; and (c) patients with preoperative CEUS and MRI examinations. Exclusion criteria were as follows: (a) patients without CEUS or MRI examination; (b) patients who failed to follow up for longer than 6 months; (c) the interval time between CEUS and MRI was more than 2 weeks; and (d) patients without surgery or biopsy.

### CEUS techniques

CEUS examination was performed in all patients who fasted for at least 8 h using a Logiq E9 ultrasonic unit (GE Healthcare, Milwaukee, WI, USA). A C1–5 2.5-MHz convex probe was used to obtain images. The periampullary lesions were first scanned by conventional ultrasound (CUS) with optimized instrument settings. Next, the patients were injected with SonoVue (Bracco, Milan, Italy) suspended in 5 mL saline using lyophilized SonoVue powder. Each patient was given 2 mL suspension through the antecubital vein within 2–3 s using a 20-G cannula, and then washed with 5 mL saline. After injection, enhanced harmonic gray-scale ultrasound, with mechanical index of 0.11 and the transmitted sound power of 100%, was used to detect the lesions around the ampulla and observe the dynamic blood perfusion of the diseased and surrounding healthy tissues. This was done at different phases post-injection, including the baseline, early phase (10–30 s after injection), and late phase (30–120 s after injection). After examination of the periampullary lesions for 2 min, the entire liver was thoroughly checked.

### MRI techniques

After 3–8 h of fasting, patients were asked to practice their breathing techniques. MRI was performed in all patients with a 3.0-T MR equipment (Philips Achieva, Holland, Netherlands) with a quasar dual gradient system and a 16.0-channel phased-array Torso coil in the supine position. Drinking water or conventional oral medicines were not restricted. The MR scan started with the localization scan, followed by a sensitivity-encoding (SENSE) reference scan. The scanning sequences were as follows: breath-hold axial dual fast field echo (dual-FFE) and high spatial resolution isotropic volume exam (THRIVE) T1-weighted imaging (T1WI), respiratory triggered coronal turbo spin echo (TSE) T2-weighted imaging (T2WI), axial fat-suppressed TSE-T2WI, single-shot TSE echo-planar imaging (EPI) diffusion-weighted imaging (DWI), and MR cholangiopancreatography (MRCP). For the dynamic contrast enhancement (DCE)-MRI, axial-THRIVE-T1WI and coronal-THRIVE-T1WI were used and 15–20 mL of contrast agent Gd-DTPA was injected through the antecubital vein at a speed of 2 mL/s. DCE-MRI was performed in 3 phases, including arterial, portal, and delayed phase and images were collected after 20 s, 60 s, and 180 s, respectively. To eliminate interference of the signal from fat, fat-saturation inhibition was used in T2WI.

### Pathological examination

The pathological data from all of the cases were analyzed by two pathologists with more than 15 years of experience. The pathologists were blinded to the clinical and imaging findings.

### Imaging analysis

All CUS and CEUS images were independently analyzed by two physicians with more than 10 years of diagnostic experience in abdominal ultrasonography. The criteria for detecting PAC by CUS and CEUS included a low, equal, or high echogenicity lump, along with perfusion. The scans were reviewed to determine the presence of PAC features. If there was a discrepancy between the two physicians, an agreement was reached by consensus.

Post-processing of images was performed using the Extended MR Workspace R2.6.3.1 (Philips Healthcare, Hong Kong, China) with the FuncTool package. All imaging examinations and measurements were performed on the workstation by two experienced radiologists who were blinded from the clinical and pathological findings, and evaluation was performed by the same observational items and criteria. Disagreements over the findings between the two radiologists were resolved by consensus. MRI showed typical PAC imaging manifestations: (1) the mass was nodular or invasive; (2) liver parenchyma on T1WI was equal or marginally lower; (3) liver parenchyma on T2WI was equally or slightly stronger, and fat-saturation was inhibited; (4) DWI showed high tension; (5) the mass was mild or moderate enhancement after contrast; and (6) when MRCP was performed, the bile duct suddenly terminated asymmetrically and expanded proportionally (double-duct signs may occur when the lesion obstructed the ducts) [7, 17, 18].

### Statistical analysis

Continuous variables were described as mean ± standard deviation (SD) or median and range. Categorical variables were represented as the absolute and relative frequencies. The sensitivity, specificity, positive predictive value (PPV), negative predictive value (NPV), and accuracy of MRI, CEUS and CCWM were calculated and compared. The data were analyzed using SPSS 23.0 (IBM, Chicago, IL, USA). Comparisons between groups were conducted using Fisher’s exact probability test for categorical data. When all theoretical numbers (T) ≥ 5 and the total sample size *n* ≥ 40, the Pearson’s chi-square test was used. When the theoretical number 1 ≤ T < 5, and the total sample size n ≥ 40, the continuity-adjusted chi-square test was adopted. If there is a theoretical number T < 1 or a total sample size *n* < 40, the Fisher’s exact test was used. For all analyses, *p*-values < 0.05 were considered statistically significant.

## Results

### General information

As shown in Table 1, 102 patients (50 males, 52 females; age range, 25–75 years; BMI, 20.84 ± 2.54 Kg/m^2^) who had undergone both MRI and CEUS in our hospital between June 1, 2015 and May 1, 2018 were enrolled. The patients were admitted due to jaundice (81/102), abdominal pain (67/102), diarrhea (12/102), abdominal distension (35/102), and vomiting (9/102). Among them, 74 cases had malignant lesions, including 20, 11, 12, and 31 carcinomas of the lower CBD, ampulla, duodenum and pancreatic head, respectively. Twenty-eight cases were benign lesions, including nine chronic mass pancreatitis, nine inflammation stenosis of CBD, and ten duodenum papilla inflammation. There were 75 patients who underwent radical PD. Additionally, 27 patients underwent endoscopic ultrasonography (EUS) or endoscopic retrograde cholangiopancreatography (ERCP). Among the 102 PSOL cases, the average lesion diameter ranged from 0.7 to 6.7 cm (median 2.25 cm).

**Table 1:** Clinical characteristics and laboratory findings of enrolled patients with PSOLs

Variable	Median Value (range)
Age (years)	56 (25–75)
BMI (Kg/m^2^)	20.55 (16.81–26.04)
White Blood Cell (10^9^/L)	6.23 (2.76–19.86)
Neutrophil (10^9^/L)	4.09 (1.39–16.50)
Erythrocyte (10^9^/L)	4.14 (2.36–5.20)
Hemoglobin (g/L)	120 (58–152)
Platelet (10^9^/L)	216.5 (112.0–574.0)
Total Bilirubin (μmol/L)	21.7 (5.8–327.4)
Direct Bilirubin (μmol/L)	13.3 (1.5–230.4)
Total Protein (g/L)	62.7 (12.8–92.6)
Albumin (g/L)	36.7 (7.5–56.1)
Alpha Fetoprotein (ng/ml)	3.20 (1.23–22.58)
Carbohydrate Antigen 19–9 (U/ml)	86.12 (1.15–400.53)
Carbohydrate Antigen 12–5 (U/ml)	13.61 (1.58–59.70)
Carcinoembryonic Antigen (ng/ml)	3.40 (1.23–59.51)

### Detection of PSOLs by MRI, CEUS, and CCWM

From the 102 PSOL patients, 94, 93, and 101 cases were accurately detected by CEUS, MRI, and CCWM, respectively. The cases missed or misdiagnosed by CEUS and MRI are shown in Tables 2 and 3. There was one case misdiagnosed by CCWM, which was inflammation stenosis of the CBD misdiagnosed as duodenal papillary carcinoma.

**Table 2:** Results that were misdiagnosed or missed by magnetic resonance imaging (MRI)

MRI diagnostic results	Pathological results	Total
Chronic mass pancreatitis	Pancreatic carcinoma	2
Common bile duct stone	Common bile duct carcinoma	3
No positive finding	Duodenal papillary carcinoma	1
Biliary surgery change	Common bile duct carcinoma	1
Common bile duct stone	Duodenal papillary carcinoma	1
Common bile duct inflammation stenosis	Pancreatic carcinoma	1

**Table 3:** Results that were misdiagnosed by contrast-enhanced ultrasound (CEUS)

CEUS diagnostic results	Pathological results	Total
Duodenal papillitis	Duodenal papillary carcinoma	3
No positive finding	Common bile duct carcinoma	1
Chronic mass pancreatitis	Pancreatic carcinoma	2
Pancreatic pseudocyst	Pancreatic carcinoma	1
Duodenal papillary carcinoma	Common bile duct inflammation stenosis	1

As shown in Table 4, the specificity, PPV, and accuracy of CCWM were more significantly effective than MRI or CEUS individually (all *p* < 0.05). However, there were no significant differences in the sensitivity or NPV between the three diagnostic methods (all *p* > 0.05). Differences in the specificity, PPV, and accuracy between the MRI and CEUS groups were not statistically significant (all *p* > 0.05).

**Table 4:** Detection of PSOLs with MRI, CEUS, and combined CCWM

Modality	Sensitivity	Specificity	PPV	NPV	Accuracy
MRI	100% (65/65)	75.68% (28/37)	87.83% (65/74)	100% (28/28)	91.17% (93/102)
CEUS	98.53% (67/68)	79.41% (27/34)	90.54% (67/74)	96.42% (27/28)	92.15% (94/102)
MRI + CEUS	98.66% (74/75)	100% (27/27)	100% (74/74)	96.42% (27/28)	99.01% (101/102)
*P* (MRI vs. CEUS)	0.326	0.781	0.597	0.313	0.800
*P* (MRI vs. CCWM)	0.350	0.016	0.006	0.313	0.009
*P* (CEUS vs. CCWM)	0.944	0.036	0.020	NA	0.041

PPV- negative predictive value NPV- negative predictive value, MRI- magnetic resonance imaging, CEUS- contrast-enhanced ultrasound, CCWM- combination of MRI and CEUS

## Discussion

Pancreaticoduodenectomy (PD) is the standard treatment for patients with resectable PAC [19]. The median survival time for patients who undergo surgical resection is approximately 25 months [19, 20]. However, complications such as pancreatic fistulas, biliary fistulas, infections, and bleeding often appear after PD surgery. A previous study reported that the incidence of PD postoperative complications may be as high as 30–65% [21]. For patients with benign lesions, unnecessary PDs could lead to surgical complications in patients, such as pancreatic fistulas, delay gastric emptying, bile leak and bleeding, and even death in some cases. However, if malignant lesions are misdiagnosed as benign lesions, it will undoubtedly delay the treatment of the patient, which could result in death. Currently, EUS and ERCP display high diagnostic value for ampullary lesions, yet both modalities are highly invasive and present with high complication rates. For small lesions, repeated punctures for biopsy may be required, which can be painful for the patient [4, 14, 22]. Therefore, it is better for physicians to accurately diagnose PSOLs using non-invasive methodologies preoperatively.

**Fig. 1:**
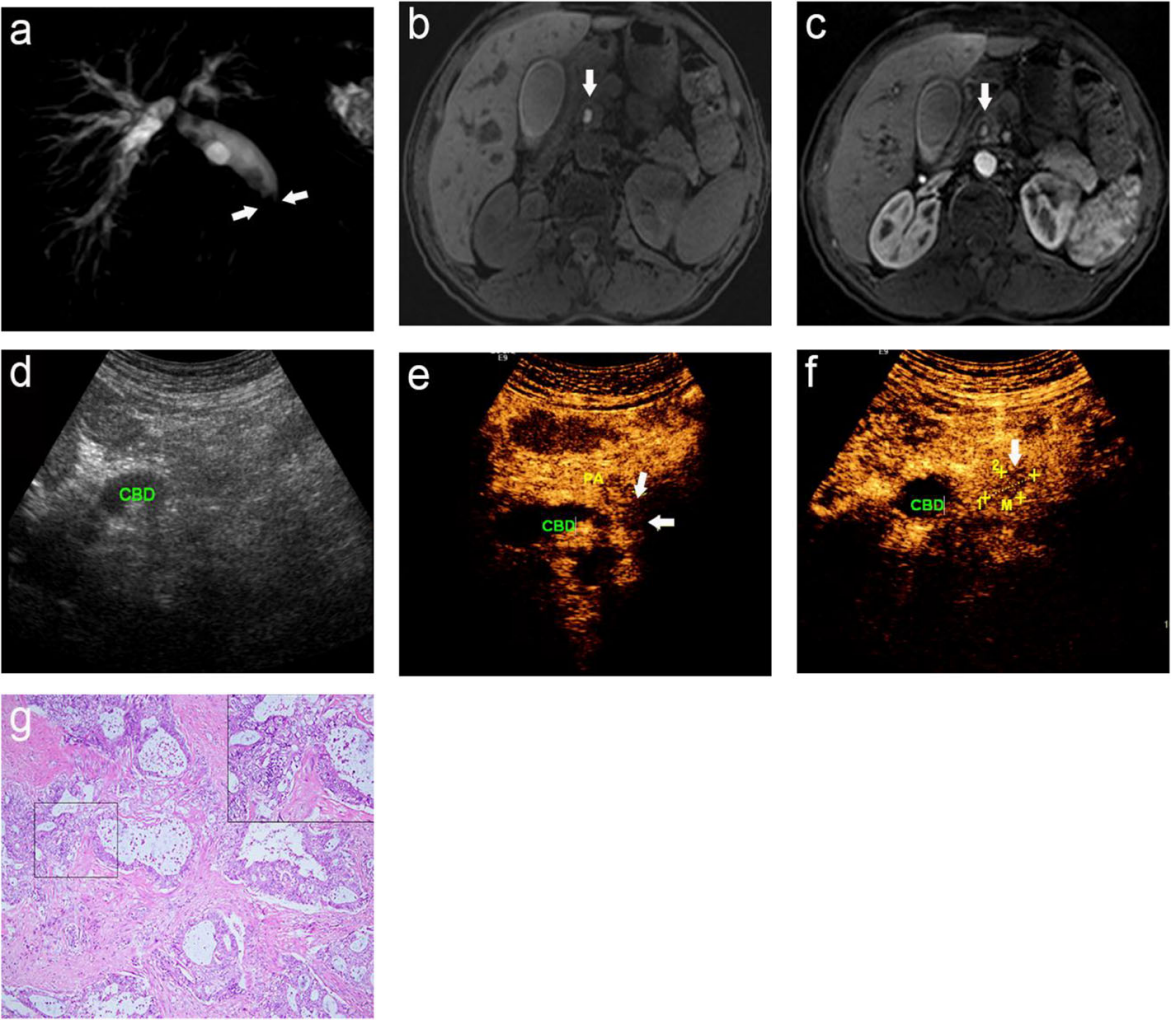
A 58-year-old male was misdiagnosed as having a common bile duct stone by MRI, which was pathologically-confirmed as choledochal adenocarcinoma; (**a**) MRCP image displays the narrowing (arrow) of the distal CBD suddenly and the expansion of the biliary tract; (**b**) Axial T1-weighted images and (**c**) T1-weighted DCE-MRI show high signal nodules in the CBD, and the possibility of stones was considered (arrow), yet no enhancement is seen in the structure of bile duct wall; (**d**) CUS shows dilated CBD; (**e**, **f**) CEUS shows a cauliflower-like mass with uneven enhancement (arrow), which should be considered for common bile duct carcinomas; (**g**) Histopathological examination reveals that some malignant glands infiltrated into the bile duct wall. CBD: common bile duct; PA: pancreas

In previous studies, MRI plus MRCP has shown a good performance for the differential diagnosis of malignant and benign lesions in PSOL patients. MRCP images provide anatomical information of the bile duct and pancreatic duct [23] and MR images can distinct between ampullary and periampullary carcinomas [24]. Meanwhile, MRCP can show the morphology of the bile and pancreatic ducts, and MRI can uncover periductal masses and pancreatic parenchymal abnormalities, which improves the specificity of MRCP [25, 26]. In our study, the diagnostic accuracy for PSOLs by MRI was 91.17%, which is similar to a previous study [6]. The similarity may be attributed to DCE-MRI, which can quantify the blood flow, microvascular, and capillary permeability of tumors [27]. Meanwhile, DWI can provide information about the biological structures and functional locations at the cellular and molecular levels, which is superior to that offered by conventional MRI [28]. All of the cases misdiagnosed by MRI included one tumor smaller than 2 cm in diameter and four combined with bile duct stones. This could affect the recognition and differentiation of periampullary lesions by MRI to some extent (Fig. 1). Additionally, one patient was admitted for acute suppurative obstructive cholangitis. In DWI, acute inflammation, such as the inflammation caused by papillitis, may also exhibit increased cell density, decreased extracellular space, and restricted movement of water molecules, resulting in limited diffusion, which mimics the characteristics of malignant tumors [7].

The application of CEUS in PSOL detection is as effective as MRI. CEUS can accurately detect the location and invasion range of periampullary malignant lesions, including duodenal, vascular invasion, and intrahepatic metastasis [29]. CEUS can also be used to identify non-neoplastic obstructive lesions without enhancement, especially when the tumor coexists with biliary mud, which is of great clinical value [15, 30]. Compared with MRI, CEUS is a more cost-effective strategy that is convenient for the bedside. However, CEUS cannot intuitively display biliary obstructions before surgery, and it is not suited for surgeons to make accurate surgical decisions. In addition, gastrointestinal gas also affects the quality of CEUS images. Eight patients were misdiagnosed as having PSOLs. Among these patients, one was obese, and the severe attenuation of the ultrasound beam made the diagnosis difficult. The second patient was treated with endoscopic retrograde cholangiopancreatography (ERCP) for cholelithiasis, which lead to normal anatomical variations. Four patients were confirmed as having lower cholangiocarcinoma complicated with cholesterol stones. Hyperechoic calculi and its accompanying acoustic shadow made the diagnosis of PAC difficult. The other two pancreatic carcinoma were misdiagnosed as a pancreatic pseudocyst, which was attributed to the patient having a history of biliary pancreatitis 2 months prior to imaging.

**Fig. 2:**
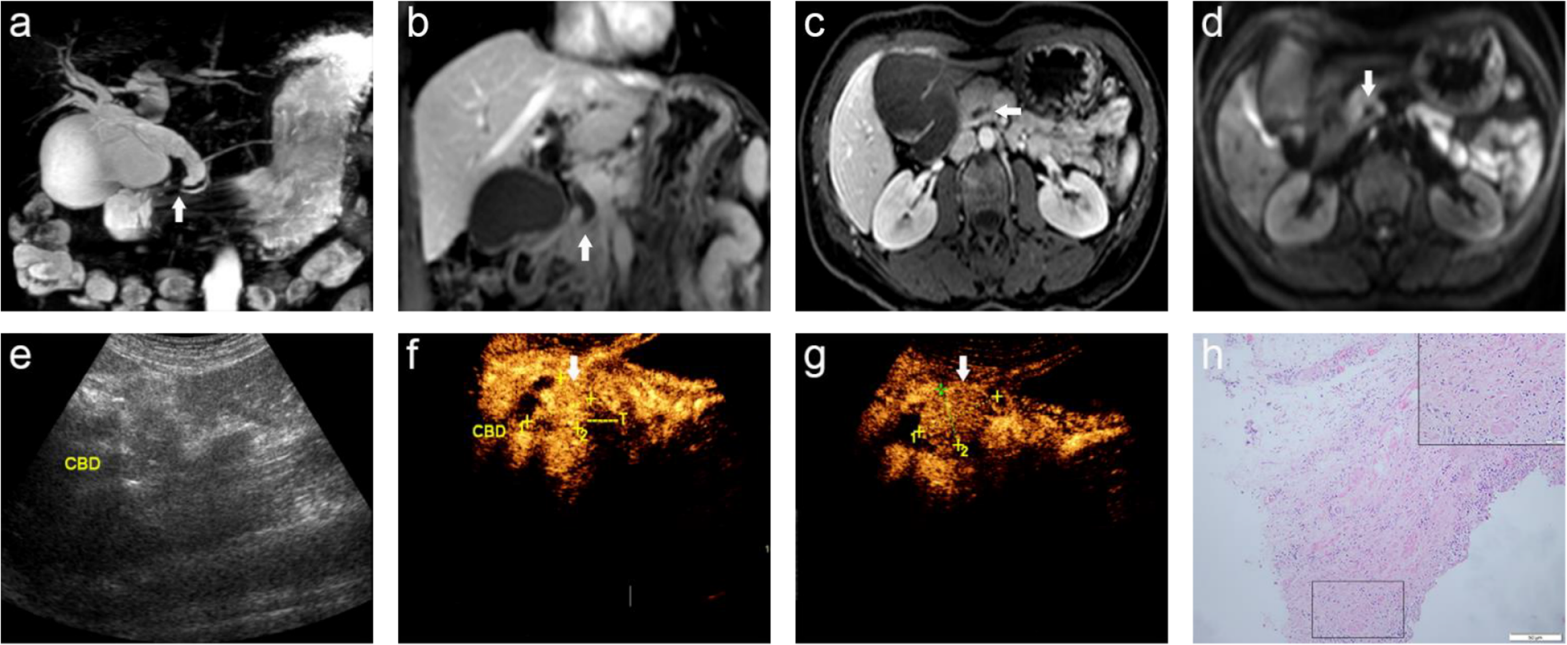
A 56-year-old female with common bile duct inflammation stenosis was misdiagnosed as having duodenal papillary carcinoma by CEUS; (**a**) MRCP image shows dilation of the intrahepatic and extrahepatic bile ducts with the lower part of the CBD tapering gradually (arrow) and the gallbladder increasing significantly. **b** Coronary contrast-enhanced T1WI shows the lower part of the CBD gradually becoming thin and occlusive (arrow) with no definite sign of mass-occupying lesions in the travel area of the intrahepatic and extrahepatic bile ducts; (**c**) Axial T1-weighted DCE-MR image shows the obstruction plane is in the lower part of the CBD (arrow), and the possibility of inflammatory stenosis and occlusion is considered. **d** DWI shows no abnormal signal is found; (**e**) CUS displays the choledochoectasia; (**f**, **g**) Uneven and highly intensified duodenal papilla region (arrow), and duodenal papillary carcinoma is considered; (**h**) Histopathological examination shows proliferative fibrous connective tissue of the bile duct wall, and the hyperplastic bile duct epithelium can be seen in the deep layer of the bile duct wall. CBD: common bile duct

Due to the limitations of MRI and CEUS individually, CCMW were used to detect PSOLs in this study. The findings demonstrate that CCWM provides higher diagnostic accuracy and improved sensitivity than MRI or CEUS individually for the detection of PSOL. There was only one case misdiagnosed by CCWM, which was treated with ERCP for cholelithiasis 1 month before this study and the normal anatomy changed by the surgery, which further increased the difficulty of the diagnosis (Fig. 2). CCWM can not only help better detect and identify PSOLs but can also avoid the shortcomings of MRI and CEUS examinations individually, which aids in the clinical diagnosis and treatment of PSOLs. CCWM improved the ability to identify benign and malignant PSOL, reduced the occurrence of unnecessary PD caused by benign lesions, and allowed for the timely and effective treatment for those malignant diseases.

There are several limitations of this study. First, the number of patients with benign lesions was small, suggesting that additional data are needed to verify our findings. Secondly, only patients that underwent surgery or biopsy in a single hospital were enrolled. Although the diagnostic physicians were blinded to the endoscopic, surgical, and histopathologic findings of patients, they did know that the tumor existed. Finally, this study was a retrospective analysis, and a randomized controlled study should be performed in the future.

## Conclusions

CCWM shows improved diagnostic accuracy and specificity for the detection of PSOLs in comparison to MRI and CEUS individually, which is of high clinical value for distinguishing between patients with benign or malignant PSOLs. The accurate and rapid assessment of patients with PSOLs is essential for optimizing treatment strategies.

## Data Availability

The datasets generated and analyzed during the current study are available from the corresponding author on reasonable request.

## Abbreviations

CBD: common bile duct
CEUS: contrast-enhanced ultrasound
CUS: conventional ultrasound
DCE-MRI: dynamic contrast-enhanced magnetic resonance imaging
dual-FFE: dual fast field echo
DWI: diffusion-weighted imaging
EPI: echo-planar imaging
ERCP: endoscopic retrograde cholangiopancreatography
EUS: endoscopic ultrasonography
MRCP: magnetic resonance cholangiopancreatography
MRCP: MR cholangiopancreatography
MRI: magnetic resonance imaging
PAC: periampullary cancer
PD: pancreaticoduodenectomy
PSOL: periampullary space-occupying lesion
TSE: turbo spin echo

## References

[1] Berberat PO, Kunzli BM, Gulbinas A, Ramanauskas T, Kleeff J, Muller MW (2009). An audit of outcomes of a series of periampullary carcinomas. Eur J Surg Oncol.

[2] Baghmar S, Agrawal N, Kumar G, Bihari C, Patidar Y, Kumar S, et al. Prognostic factors and the role of adjuvant treatment in Periampullary carcinoma: a single-Centre experience of 95 patients. J Gastrointest Cancer. 2018. 10.1007/s12029-018-0058-7 29464529

[3] Hashemzadeh Shahryar, Mehrafsa Behzad, Kakaei Farzad, Javadrashid Reza, Golshan Rosa, Seifar Fatemeh, Hajibonabi Farid, Salmannezhad Khorami Farzad (2018). Diagnostic Accuracy of a 64-Slice Multi-Detector CT Scan in the Preoperative Evaluation of Periampullary Neoplasms. Journal of Clinical Medicine.

[4] Puli SR, Singh S, Hagedorn CH, Reddy J, Olyaee M (2007). Diagnostic accuracy of EUS for vascular invasion in pancreatic and periampullary cancers: a meta-analysis and systematic review. Gastrointest Endosc.

[5] Chang S, Lim JH, Choi D, Kim SK, Lee WJ (2008). Differentiation of ampullary tumor from benign papillary stricture by thin-section multidetector CT. Abdom Imaging.

[6] Zhang T, Su ZZ, Wang P, Wu T, Tang W, Xu EJ (2016). Double contrast-enhanced ultrasonography in the detection of periampullary cancer: comparison with B-mode ultrasonography and MR imaging. Eur J Radiol.

[7] Lee NK, Kim S, Seo HI, Kim DU, Woo HY, Kim TU (2013). Diffusion-weighted MR imaging for the differentiation of malignant from benign strictures in the periampullary region. Eur Radiol.

[8] Li B, Zhang L, Zhang ZY, Ni JM, Lu FQ, Wu WJ (2015). Differentiation of noncalculous periampullary obstruction: comparison of CT with negative-contrast CT cholangiopancreatography versus MRI with MR cholangiopancreatography. Eur Radiol.

[9] Zhang TT, Wang L, Liu HH, Zhang CY, Li XM, Lu JP (2017). Differentiation of pancreatic carcinoma and mass-forming focal pancreatitis: qualitative and quantitative assessment by dynamic contrast-enhanced MRI combined with diffusion-weighted imaging. Oncotarget..

[10] Zhong L (2007). Magnetic resonance imaging in the detection of pancreatic neoplasms. J Dig Dis.

[11] Wang FB, Ni JM, Zhang ZY, Zhang L, Wu WJ, Wang D (2014). Differential diagnosis of periampullary carcinomas: comparison of CT with negative-contrast CT cholangiopancreatography versus MRI with MR cholangiopancreatography. Abdom Imaging.

[12] D'Onofrio M, Zamboni G, Faccioli N, Capelli P, Pozzi MR (2007). Ultrasonography of the pancreas. 4. Contrast-enhanced imaging. Abdom Imaging.

[13] Zhou X, Liu JB, Luo Y, Yan F, Peng Y, Lin L (2010). Characterization of focal liver lesions by means of assessment of hepatic transit time with contrast-enhanced US. Radiology..

[14] Zhou X, Luo Y, Peng YL, Cai W, Lu Q, Lin L (2011). Hepatic perfusion disorder associated with focal liver lesions: contrast-enhanced US patterns--correlation study with contrast-enhanced CT. Radiology..

[15] Taimr P, Jongerius VL, Pek CJ, Krak NC, Hansen BE, Janssen HL (2015). Liver contrast-enhanced ultrasound improves detection of liver metastases in patients with pancreatic or Periampullary Cancer. Ultrasound Med Biol.

[16] Dietrich CF, Ignee A, Trojan J, Fellbaum C, Schuessler G (2004). Improved characterisation of histologically proven liver tumours by contrast enhanced ultrasonography during the portal venous and specific late phase of SHU 508A. Gut..

[17] Sugita R, Furuta A, Ito K, Fujita N, Ichinohasama R, Takahashi S (2004). Periampullary tumors: high-spatial-resolution MR imaging and histopathologic findings in ampullary region specimens. Radiology..

[18] Wu DS, Chen WX, Wang XD, Acharya R, Jiang XH (2012). Pancreaticobiliary duct changes of periampullary carcinomas: quantitative analysis at MR imaging. Eur J Radiol.

[19] Winter JM, Brennan MF, Tang LH, D'Angelica MI, Dematteo RP, Fong Y (2012). Survival after resection of pancreatic adenocarcinoma: results from a single institution over three decades. Ann Surg Oncol.

[20] El Nakeeb A, El Shobary M, El Dosoky M, Nabeh A, El Sorogy M, El Eneen AA (2014). Prognostic factors affecting survival after pancreaticoduodenectomy for pancreatic adenocarcinoma (single center experience). Hepatogastroenterology..

[21] Hill JS, Zhou Z, Simons JP, Ng SC, McDade TP, Whalen GF (2010). A simple risk score to predict in-hospital mortality after pancreatic resection for cancer. Ann Surg Oncol.

[22] Andriulli A, Loperfido S, Napolitano G, Niro G, Valvano MR, Spirito F (2007). Incidence rates of post-ERCP complications: a systematic survey of prospective studies. Am J Gastroenterol.

[23] Andersson M, Kostic S, Johansson M, Lundell L, Asztely M, Hellstrom M (2005). MRI combined with MR cholangiopancreatography versus helical CT in the evaluation of patients with suspected periampullary tumors: a prospective comparative study. Acta Radiol.

[24] Kim TU, Kim S, Lee JW, Woo SK, Lee TH, Choo KS (2008). Ampulla of Vater: comprehensive anatomy, MR imaging of pathologic conditions, and correlation with endoscopy. Eur J Radiol.

[25] Kim MJ, Mitchell DG, Ito K, Outwater EK (2000). Biliary dilatation: differentiation of benign from malignant causes--value of adding conventional MR imaging to MR cholangiopancreatography. Radiology..

[26] Nikolaidis P, Hammond NA, Day K, Yaghmai V, Wood CG, Mosbach DS (2014). Imaging features of benign and malignant ampullary and periampullary lesions. Radiographics..

[27] Huang XQ, Shu J, Luo L, Jin ML, Lu XF, Yang SG (2016). Differentiation grade for extrahepatic bile duct adenocarcinoma: Assessed by diffusion-weighted imaging at 3.0-T MR. Eur J Radiol.

[28] Zhang L, Tang M, Min Z, Lu J, Lei X, Zhang X (2016). Accuracy of combined dynamic contrast-enhanced magnetic resonance imaging and diffusion-weighted imaging for breast cancer detection: a meta-analysis. Acta Radiol.

[29] Alrashed A, Ahmad H, Khalili K, Kim TK, Jang HJ, Atri M (2018). Negative predictive value of contrast-enhanced ultrasound in differentiating avascular solid-appearing from vascularized masses: a retrospective consecutive study. J Ultrasound Med.

[30] Sparchez Z, Radu P (2014). Role of contrast enhanced ultrasound in the assessment of biliary duct disease. Med Ultrason.

